# The effectiveness and acceptability of the Bergen 4-day treatment for adolescents with OCD: a replication and extension

**DOI:** 10.1186/s12888-024-05601-w

**Published:** 2024-02-21

**Authors:** Solvei Harila Skjold, Kristen Hagen, Michael G. Wheaton, Kay Morten Hjelle, Thröstur Björgvinsson, Bjarne Hansen

**Affiliations:** 1 Bergen Center for Brain Plasticity, Haukeland University Hospital, Bergen, Norway; 2 Center for Crisis Psychology, Faculty of Psychology, University of Bergen, Bergen, Norway; 3 OCD-team, Haukeland University Hospital, Bergen, Norway; 4 Møre and Romsdal Hospital Trust, Hospital of Molde, Molde, Norway; 5 Department of Psychology, Barnard College, Columbia University, New York, USA; 6 Center for OCD and Related Disorders, Massachusetts General Hospital, Boston, USA; 7 Department of Psychiatry, Harvard Medical School, Boston, USA; 8 Behavioral Health Partial Hospital Program, McLean Hospital, Belmont, USA; 9 Department of Mental Health, Norwegian University of Science and Technology, Trondheim, Norway

**Keywords:** OCD, Adolescents, Concentrated treatment, Exposure, B4DT, CBT

## Abstract

**Background:**

B4DT is a concentrated treatment format with prolonged sessions of exposure and ritual prevention (ERP) delivered over four consecutive days. Two previous open trials demonstrated promising results of the Bergen 4-day treatment (B4DT) for adolescents with obsessive-compulsive disorder (OCD). The aim of the current study was to replicate the initial results with a new sample of adolescents and different therapists at different sites across Norway.

**Methods:**

Forty-three youths participated in treatment program. At pretreatment, posttreatment, and the three-month follow-up, OCD symptoms were assessed using the CY-BOCS interview, while the GAD-7 and PHQ-9 were administered to rate general anxiety symptoms and depressive symptoms. Acceptability and patient satisfaction with the treatment were rated with the CSQ-8.

**Results:**

All symptoms were significantly reduced at posttreatment and follow-up. At posttreatment, 36 patients (85.71%) were defined as responders, while 29 patients (69.05%) achieved remission. At the three-month follow-up, 36 patients (92.3%) were defined as responders, while 33 patients (84.62%) were in remission. CSQ-8 scores indicated that the patients were highly satisfied with the treatment.

**Conclusions:**

The B4DT was successfully replicated in a new sample at different sites across Norway, which indicates that this treatment is generalizable, effective and acceptable to adolescents with OCD.

## Background

The defining features of obsessive-compulsive disorder (OCD) are recurrent unwanted obsessive thoughts coupled with compulsive behaviour that functions to reduce the anxiety and distress caused by these thoughts. In individuals with OCD, obsessions and compulsions are time-consuming and significantly impair daily functioning. Functioning at school, at home, socially, and during spare time activities may be substantially affected [1, 2]. Often, OCD affects not only the person with OCD but also their whole family [1]. More than 50% of individuals with OCD started having OCD symptoms during childhood or adolescence [3, 4]. Without treatment, spontaneous recovery is rare [5]. The prevalence in adolescents is approximately 1–3%, while the prevalence is lower in younger children [6, 7].

Cognitive behavioural therapy (CBT) consisting of exposure and response prevention (ERP) is an effective treatment for children and adolescents with OCD, as documented in numerous open and randomized controlled trials [8–11]. ERP has been successfully delivered in different formats, including individual therapy, group therapy, and family-based therapy, and at different delivery schedules, including weekly therapy and more intensive therapy [12–15]. Different forms of brief, intensive and concentrated CBT treatments for OCD and other conditions involving pathological anxiety have been developed during the last two decades, with comparable results in regard to remission and recovery posttreatment and lower attrition rates compared to standard delivery models [16, 17].

Delivering more intensive treatment within a shorter time span, rather than weekly therapy delivered across a longer time span, has the potential advantage of helping patients recover faster [18]. Concentrated treatment also has the benefit of allowing a patient and their therapist to intensively practice exposure to different triggers, including in the patient’s typical day-to-day environment [18]. The Bergen 4-Day treatment (B4DT) was developed to implement ERP treatment for OCD patients in an intensive format, delivering the entire treatment within four consecutive days [19]. Since then, this approach to treatment has been documented to be effective in treating OCD patients within different samples and at different sites [20–22].

Research on concentrated CBT for adolescents with OCD is limited [16]. Two previous effectiveness studies of concentrated treatment using a child and adolescent version of the B4DT reported promising results for adolescents in Bergen Norway [18, 23]. However, these past results are limited to a single centre with a small team of therapists. Therefore, in the present study, we aimed to see if the results from the two previous studies in Bergen could be replicated in a new sample with new therapists at different sites across Norway. In addition, the prior trials included a broader age range of youth. Since there are fewer studies specific to the 16-18-year-old age group [24], the current study focused on youth of this age. This specific age range warrants particular attention given that these teenage years can be turbulent. Adolescents at this age may have different preferences and treatment needs compared to younger children, youths in their early teenage years and adults [24, 25]. Some previous studies found that older age among children and adolescents could be a predictor of poorer treatment outcome [8, 26], but the findings are not consistent [8, 27]. Limiting the study to 16-18-year-olds was a way to reduce variability in age and increase the focus of our research.

In addition to determining whether treatment helps reduce symptoms of OCD, it is also important to determine whether the treatment is acceptable to these adolescent patients. This is particularly important for this population given that on average, adolescents attend fewer than half of their mental health care visits [28, 29]. Engaging adolescents in mental health treatment is increasingly recognized as an important challenge [30]; therefore, it is of paramount importance to determine effective treatments that are acceptable to teenagers so that they will be willing to engage in their treatment. Past studies of the acceptability of the B4DT showed that this treatment is highly acceptable and evidenced high ratings of client satisfaction with the treatment [19, 22], although these data have only been reported in adults. Client satisfaction with the B4DT was not reported in past studies with youth samples [18, 23], so whether the B4DT is acceptable to adolescents remains an important unanswered question. Therefore, the present study also included the Client Satisfaction Questionnaire (CSQ-8) to assess acceptability.

The aim of the current study was to investigate the effectiveness and acceptability of the B4DT delivered to adolescents with OCD at different clinics. This study was an open trial effectiveness study, a replication study with an extension, and part of a quality study documenting the results of OCD treatment at public specialized OCD treatment clinics across Norway. We hypothesized that the treatment could be replicated with similar results with different samples, different therapists, and at new sites in Norway. We also expected the B4DT to be acceptable to adolescents.

## Methods

### Design and participants

The current study was a naturalistic open trial effectiveness study that included the actual patients referred to specialized outpatient OCD treatment units in Norway. The patients were recruited between January 2020 and December 2022. A total of 43 patients from seven different specialized outpatient OCD treatment units across Norway were included. At first, 44 patients were included, but one patient was later excluded from the study, as the therapists discovered that the patient did not have OCD.All participants in the present study were 16–18 years old and referred to child and adolescent OCD units in Norway for assessment and treatment. Two of the patients turned 18 a few days before the treatment started, but the rest were younger than 18 years old.

To be included in this study, patients had to be between 16 and 18 years old, diagnosed with moderate or severe OCD according to ICD-10 diagnostic criteria, and seeking OCD treatment from the public health system in Norway. They received information about the treatment, decided whether to receive the treatment, and signed a written informed consent form to participate in clinical research. Participation in the study had no impact on the treatment provided. The study was approved by the Regional Committee for Medical and Health Research Ethics of Northern Norway (REK Nord: 2023-606482).

Patients were not included in the present study if they had active psychosis, drug addiction, mania or suicidality, a severe eating disorder and a BMI that was too low, autism spectrum disorder or an intellectual disability that would make them unable to understand psychoeducation or cope with participating in a group. If patients were on pharmacotherapy before they were referred to the clinic, the dose should be stable for four weeks before the treatment.

Standardized assessments included diagnostic interviews to diagnose OCD and exclusion diagnoses, as well as clinical interviews to assess motivation, insight and expectations for the treatment.

#### Adherence and competence

All therapists were certified as B4DT OCD therapists by participating in courses and training before they were responsible for treating their own OCD patients. They also all worked in specialized OCD units for children and adolescents and used a standardized manual for B4DT OCD treatment. As the assessment and treatment occurred in regular outpatient clinics across the country, not all assessment were done independent.

### Diagnostics and assessment

The patients underwent a standard assessment procedure, including a semistructured diagnostic interview (the MINI or Kiddie-SADS) and the CY-BOCS. Both interviews assess current and lifetime psychopathology and have good psychometric properties [31, 32].

#### Primary outcome measure

The primary outcome measure was the semistructured clinician-administered interview version of the Children’s Yale-Brown Obsessive Compulsive Scale (CY-BOCS) [33]. The CY-BOCS also has good psychometric properties and has been widely used to assess OCD symptoms. It rates the severity of obsessions and compulsions based on five dimensions: time occupied by symptoms, how the symptoms interfere with daily life, distress, resistance, and control. The CY-BOCS categorizes symptom severity on a scale from 0 to 40, with divisions into subclinical symptoms (0–7), mild symptoms [8–15], moderate symptoms [16–23], severe symptoms [8, 24–30] and extreme symptoms [31–39].

#### Secondary outcome measures

The secondary outcome measures were self-report questionnaires, including the PHQ-9, GAD-7, and CSQ-8, which were filled out digitally. The PHQ-9 self-report questionnaire measures depressive symptoms based on nine criteria in the DSM-IV, with each item scored on a four-point Likert scale [34]. The GAD-7 self-report questionnaire measures symptoms of general anxiety based on criteria from the DSM-IV, with items scored on a four-point Likert scale [35]. The CSQ-8 self-report questionnaire measures client satisfaction with the treatment, with eight different questions scored from 1 (very low satisfaction) to 4 (very high satisfaction); the total score can range from 0 to 32, where 32 is the highest possible client satisfaction score [36].

### Treatment

The patients received the B4DT with 3–4 adolescent patients in each group. The youth version of the B4DT consists of delivering the entire treatment within four consecutive days and is an individual tailored treatment provided within a group format; parents are present during the treatment on Day one and Day four [18]. The patient-therapist ratio is 1:1. Day one consists of psychoeducation for the youths and their parents together; after psychoeducation, the parents and youths split up so that the youths can plan exposure tasks together and get to know each other, while the parents obtain information about how to support their child and reduce family accommodations for the child’s OCD. Days two and three are devoted to therapist-assisted exposure training, with short group meetings together with the other patients and therapists. On Day four, the treatment is summarized, some psychoeducation is repeated with an emphasis on how to maintain the results, continue to get better and prevent relapse; the patients talk about what they have learned from the therapy, and they make plans for further exposure training within the next 3 weeks. The treatment format is further described in Riise et al. [18].

### Statistical analysis

IBM SPSS Statistics (version 27) was used to perform the statistical analyses. Independent samples *t* tests were performed to investigate whether demographic variables were related to symptom severity prior to treatment. The change in symptoms over time (as measured in separate analyses by the CY-BOCS, PHQ-9 and GAD-7) was analysed with a linear mixed model (LMM) design for repeated measures, with time as a fixed effect and each patient as a random intercept, and Bonferroni corrections were used for multiple comparisons. The use of an LMM design allowed patients with missing data to be included in the analyses without imputation or listwise deletion [37, 38]. A variance component covariance structure was applied, and restricted maximum likelihood estimation with Satterthwaite approximation was chosen to estimate t- and *p* values. To investigate the change from posttreatment to follow-up, two new slope variables were created: the first represented the change from pretreatment to posttreatment and the second represented the change from posttreatment to follow-up. To investigate whether the patients who had received previous treatment differed at baseline, this variable was added as a covariate in a separate analysis, and the time*previous treatment interaction was used to investigate differences in changes over time. Cohen´s d *(M*_*1*_*– M*_*2*_*)/SD*_*pooled*_*)* was used to calculate effect sizes. The intraclass correlation was calculated to investigate whether there were site differences in the mean CY-BOCS score. One-way ANOVA and independent sample t tests were used to compare demographic variables to pre-CY-BOCS scores with listwise deletion of missing data. An independent sample t test was used to compare digital and face-to face-treatment, with listwise deletion of missing data. To benchmark the CY-BOCS scores from the present study against those from previous studies, we used independent samples t tests and calculated these based on means, SDs and the numbers of participants using a calculator.

## Results

Forty-three patients, aged 16 to 18 years, were included in the present study and analyses. The dataset had a low percentage of missing data for the CY-BOCS interview, with no patients missing data before treatment, two (4.9%) missing data for the one-week follow-up and four (9.3%) missing data for the three-month follow-up. For the self-report measures (the GAD-7 and PHQ-9), six (14%) patients were missing information before treatment, 16 (37.2%) were missing information for the one-week follow-up and 23 (53.4%) were missing information for the three-month follow-up. No patients dropped out of treatment.

### Demographic information/pretreatment characteristics

Table 1 contains an overview of the baseline patient characteristics. Patients in the current study received treatment from seven different specialized OCD treatment units across Norway, representing all health regions of Norway, except the northern part. Among these patients, 33 (76.74%) were female, and 10 (23.26%) were male. Two patients turned 18 years old a few days prior to treatment, while the rest were 16 and 17 years old. The time since the onset of the disorder ranged between one year and as long as they could remember, with a mean of 3.21 years. Thirty-two patients were not on any medication, whereas five patients took SSRIs and one patient took ADHD medication; the medication status for five patients was not reported. Twenty-two patients had not previously received any treatment for their OCD, while 21 patients had previously received treatment. Three of them reported having received CBT treatment with exposure and response prevention and one reported receiving only medication; the kind of treatment prior to the current study was not reported by the remainder of the patients. Unfortunately, data on comorbidities were not reported for 20 of the patients. Among the patients for whom comorbidities were reported, 11 (47.83%) had comorbid anxiety disorders, seven (30%) had comorbid depression, nine (39.13%) had no comorbidities, two (8.70%) had ADHD, and one (4.35%) had Asperger syndrome.

**Table 1:** Demographics and diagnostic description of the sample (*N* = 43)

Variable	M (SD)
Age (years)	16.77 (0.53)
Duration of the disorder (years)	3.21 (2.48)
	N (%)
Sex: females	33 (76.74)
Had received previous treatment	22 (51.16)
Comorbidity Not reported	20 (46.51)
No comorbidities Comorbid anxiety Comorbid depression	9 (20.93) 11 (25.58) 7 (16.28)
Psychotropic medication No medication SSRIs/SNRIs Metylphenidate Not reported	5 (11.63) 32 (74.40) 5 (11.63) 1 (2.32) 6 (13.95)

### Primary and secondary outcome measures

A summary of mean scores across the three measures can be found in Table 2, in addition to the effect size for the change. When comparing the originator site against the other sites, we found no statistically significant differences in CY-BOCS scores (ICC = 0.03). We found a statistically significant effect of time on CY-BOCS scores (b_0_ = 26.0, *p* <.001, b_1_=-14.96, *p* <.001, b_2_=-0.17.42, *p* <.001), which indicated that there was a reduction in symptoms over time/treatment. There was a statistically significant change from the posttreatment to follow-up CY-BOCS scores (b=-2.47, *p* =.01). When comparing the patients who had previously received treatment versus those who had not, we found no difference in CY-BOCS scores between patients who had and had not previously received treatment at baseline (b = 0.17, *p* =.91) or in the change at the two time intervals after treatment (group*time interactions: b_1_ = 1.32, *p* =.50; b_2_ = 0.02, *p* =.99). There were no significant differences at post or follow up CY-BOCS scores between the 9 patients who received either wholly digital or a hybrid version of the treatment, compared to those who received the treatment face-to-face (*p* =.30 post, *p* =.84 at three month follow up).

**Table 2:** Results and effect sizes of the primary and secondary outcome measures (*N* = 43)

Variable	Pre Mean (SD)	Post Mean (SD)	F-up Mean (SD)	ES Pre– post	ES Pre - f-up
CY-BOCS	26.0 (4.1)	11.1 (4.6)	8.4 (5.8)	3.43	3.52
GAD-7	11.3 (4.8)	8.6 (5.4)	6.4 (4.2)	0.54	1.09
PHQ-9	10.8 (6.0)	8.9 (5.6)	8.3 (5.4)	0.37	0.45

ES: Effect size measured by Cohen’s D.

Table 2 summarizes the results and effect sizes of the primary and secondary measures.

The secondary measure, the GAD-7, showed a significant effect of time/treatment (b_0_ = 11.34, *p* <.001, b_1_=-2.43, *p =*.004, b_2_=-.-4.00, *p* <.001). There was also a statistically significant change in GAD-7 scores from posttreatment to follow-up (b=-1.58, *p* =.011). We also found a statistically significant effect of time/treatment on the secondary measure of the PHQ-9 (b_0_ = 10.84, *p* <.001, b_1_=-1.98, *p =*.036, b_2_=-.-2.48, *p* =.02), but the change from posttreatment to follow-up was not significant (b = 0.50, *p* =.65).

### Response and remission

We used an adapted version of the international consensus criteria of response and remission of OCD from Mataix-Cols et al. [39], which defines response as a reduction of at least 35% in the CY-BOCS score and remission as a CY-BOCS score of 12 or below in addition to a reduction of at least 35% in the CY-BOCS score. In this sample, 36 patients (85.71% of patients) were defined as responders post-treatment at the one-week follow-up, while 29 patients (69.05%) were defined as being in remission. At the three-month follow-up, 36 patients (92.3%) were defined as responders, while 33 patients (84.62%) were in remission.

### Client satisfaction

A total of 29 patients completed the CSQ-8 posttreatment (see Table 3). The mean client satisfaction score was 30.03. The results indicated that the patients were highly satisfied with their treatment.

**Table 3:** Patient satisfaction as measured by the Client Satisfaction Questionnaire-8

	Mean score	1	2	3	4
Quality of service	3.55	0	1	11	17
Kind of service	3.75	0	0	7	22
Met needs	3.72	0	0	8	21
Would recommend to a friend	3.83	0	0	5	24
Amount of help	3.83	0	0	5	24
Deal with problems	3.86	0	0	4	25
Overall satisfaction	3.79	0	0	6	23
Would come back	3.69	0	0	9	20
Mean total score	30.03				

### Comparison to previous studies

The results from this study show the same trend as previous studies [18, 23], with a clinically significant reduction in symptoms from pretreatment to posttreatment and from pretreatment to follow-up. Benchmarking with the prior trials (see Table 4), shows that there is significant higher scores in the current study compared to the Riise et al. study from 2016 [18] (*p* =.007) but not compared to the study from Riise et al. from 2018 (*p* =.10). The current study have significant higher scores posttreatment compared to the Rise et al., 2018 study (*p* =.001), but not compared to the Riise et al., 2016 study *p* =.10). There were no significant differences between the current study and the Riise et al., 2016 study (*p* =.06) or the Riise et al., 2018 study (*p* =.35) at follow up.

**Table 4:** Comparison of CY-BOCS scores between the current study and previous studies

	Current study *N* = 43	Riise et al. 2016 (18) *N* = 22	Riise et al. 2018 (23) *N* = 41
Pretreatment	26.00 (4.06)	28.00 (4.06)	25.70 (3.72)
Posttreatment ES*	11.05 (4.63) 3.42	9.04 (4.98) 4.67	8.48 (4.54) 4.15
Follow-up ES **	8.36 (5.77) 3.52	5.87 (6.05) 5.20	6.87 (6.72) 3.95

Note: CY-BOCS means and SD. *ES: Effect size measured with Cohen’s D between pretreatment and posttreatment. **ES: Effect Size measured with Cohen’s D between pretreatment and follow up.

## Discussion

The current naturalistic study was a replication of the B4DT for adolescents, with an extension to different clinics in Norway. As previous studies have shown promising results for the B4DT [18, 23], more research with replications of the previous studies in new settings, with new therapists and at new sites is needed. We found a significant reduction in OCD symptoms from pretreatment to one week after treatment, which was sustained at the three-month follow-up. Symptoms of generalized anxiety and depression were also significantly reduced after treatment. The patients reported high satisfaction with the treatment as measured by the CSQ-8, indicating that the treatment was acceptable to these adolescents. Benchmarking with past studies, the current study had clinically significant higher scores on the pretreatment CY-BOCS scores compared to the Riise et al. study from 2016 [18], but not compared to the Riise et al. study from 2018 [23]. Posttreatment, the current study had significant higher scores on the CY-BOCS compared to the Riise et al. study from 2018 [23], but not compared to the CY-BOCS scores from the Riise et al. study from 2016 [18]. At 3-months follow-up there were no significant differences between the current study and the two previous studies on B4DT for OCD in adolescents. However, it should be noted that the two previous studies had samples from 12- to 18-year-olds, compared to the current study that had a sample from 16- to 18- year-olds. While most studies indicates that age is not a predictor of treatment response [27], there are studies that do point to age being a predictor of worsened treatment outcome [8, 26]. This indicate that age might have impacted the outcomes of the treatment, underscoring the importance of examining this particular age group closely. The sample sizes are also relatively small, which indicates that the direct comparisons between the studies should be made with caution. Overall, the results indicate that the treatment is a potentially effective treatment, and the results are relatively similar to the two previous studies on B4DT for OCD in adolescents, although there is a tendency towards slightly higher CY-BOCS scores in the current study.

Since this was a naturalistic study and the 16–18-year-old patients included in this study were those seeking help from clinical outpatient OCD treatment units at seven different sites in Norway, this study had high ecological validity. Thus, the current study indicated that this treatment is effective and can be implemented successfully in different clinics geographically spread across Norway. There were no dropouts in this study, which is in line with research showing that brief, intensive and concentrated treatment formats tend to have lower attrition rates [16]. It is particularly important to have a treatment that adolescents will be able to complete, given that past research has demonstrated that adolescents often have problems attending therapy sessions [28, 29]. Given this, concentrated brief treatments may be a particularly effective way to deliver treatment to adolescent samples.

Naturalistic studies have some limitations that should be acknowledged. As this was not a randomized controlled trial (RCT), we could not control for confounding third variables that may have affected the results. We do not know if some of these patients received booster sessions in addition to the four-day treatment. We also do not know how many patients were assessed but not included in the treatment, since we only have data from the patients included in groups from the different sites in the quality register. Another limitation is that although we pursued to secure independent assessments, not all assessment were done independent. Although all patients completed treatment, there were missing data, especially in regard to comorbidities and self-report questionnaires, and we do not know if the patients with missing data differed from those without missing data. Some of the unknown factors that we were not able to control for include the pandemic times, developmental factors or challenges specific to the developmental phase of 16-18-year-olds, differences between different therapists, etc. In addition, the present study extended the B4DT to new clinics within Norway, but whether this form of treatment is effective and acceptable to adolescents in other countries remains to be seen.

Further research about treating adolescents with OCD is needed, controlling for more factors than we were able to control for in this study. We did not include youths who were less than 16 years old in this study. Therefore, further research is needed to determine whether the results from previous studies in Bergen can also be replicated for younger children and adolescents at new sites. Further studies should include data on long-term follow up, and look closer at predictors of outcome. Further qualitative research is essential to comprehensively explore and understand the treatment experiences within this specific age demographic.

## Conclusion

This current study showed that results from previous studies of the B4DT could be replicated in a different sample, with new therapists at different sites, and in different clinics. We therefore conclude that this treatment seems to be both acceptable and effective in the treatment of OCD in adolescents. Further research is needed to determine whether this is also the case when controlling for more factors, which we were unable to control for in this study, and if the results remain the same in an RCT and with long-term follow-up. Finally, future studies are needed to verify the acceptability and effectiveness of the B4DT in international samples of adolescents with OCD.

## Data Availability

The anonymized datasets used during the current study are available from the corresponding author upon reasonable request.

## Abbreviations

B4DT: The Bergen 4-Day Treatment
GAD-7: General Anxiety Disorder − 7
CBT: Cognitive Behavioral Therapy
CY-BOCS: Children’s Yale-Brown Obsessive Compulsive Scale
CSQ-8: Client Satisfaction Questionnaire − 8
ERP: Exposure and Response Prevention
OCD: Obsessive Compulsive Disorder
PHQ-9: Patient Health Questionnaire − 9
RCT: Randomized Controlled Trial
